# The human-specific duplicated α7 gene inhibits the ancestral α7, negatively regulating nicotinic acetylcholine receptor-mediated transmitter release

**DOI:** 10.1016/j.jbc.2021.100341

**Published:** 2021-01-28

**Authors:** Carolina Martín-Sánchez, Eva Alés, Santiago Balseiro-Gómez, Gema Atienza, Francisco Arnalich, Anna Bordas, José L. Cedillo, María Extremera, Arturo Chávez-Reyes, Carmen Montiel

**Affiliations:** 1 Department of Pharmacology and Therapeutics, Medical School, Universidad Autónoma de Madrid, Madrid, Spain; 2 Department of Medical Physiology and Biophysics, Medical School, Universidad de Sevilla, Sevilla, Spain; 3 Internal Medicine Service, University Hospital La Paz-IdiPAZ, Madrid, Spain; 4 Medical School, Universidad Finis Terrae, Santiago de Chile, Chile

**Keywords:** human-specific duplicate genes, human genetics, *CHRFAM7A*, dupα7 nicotinic subunit, α7 nicotinic acetylcholine receptors, neurotransmitter release, calcium intracellular release, central nervous system, α7-nAChR, α7 subtype of the nicotinic acetylcholine receptor, ACh, acetylcholine, [Ca^2+^]_i_, intracellular concentration of Ca^2+^, CNS, central nervous system, DA, dopamine, dupα7, partial duplicated isoform of the human α7 nicotinic subunit, FRET, Föster resonance energy transfer, NSCLC, non-small-cell lung cancer, PAM, positive allosteric modulator of α7-nAChR, qPCR, real-time quantitative polymerase chain reaction, SD, standard deviation

## Abstract

Gene duplication generates new functions and traits, enabling evolution. Human-specific duplicated genes in particular are primary sources of innovation during our evolution although they have very few known functions. Here we examine the brain function of one of these genes (*CHRFAM7A*) and its product (dupα7 subunit). This gene results from a partial duplication of the ancestral *CHRNA7* gene encoding the α7 subunit that forms the homopentameric α7 nicotinic acetylcholine receptor (α7-nAChR). The functions of α7-nAChR in the brain are well defined, including the modulation of synaptic transmission and plasticity underlying normal attention, cognition, learning, and memory processes. However, the role of the dupα7 subunit remains unexplored at the neuronal level. Here, we characterize that role by combining immunoblotting, quantitative RT-PCR and FRET techniques with functional assays of α7-nAChR activity using human neuroblastoma SH-SY5Y cell variants with different dupα7 expression levels. Our findings reveal a physical interaction between dupα7 and α7 subunits in fluorescent protein-tagged dupα7/α7 transfected cells that negatively affects normal α7-nAChR activity. Specifically, in both single cells and cell populations, the [Ca^2+^]_i_ signal and the exocytotic response induced by selective stimulation of α7-nAChR were either significantly inhibited by stable dupα7 overexpression or augmented after silencing *dupα7* gene expression with specific siRNAs. These findings identify a new role for the dupα7 subunit as a negative regulator of α7-nAChR-mediated control of exocytotic neurotransmitter release. If this effect is excessive, it would result in an impaired synaptic transmission that could underlie the neurocognitive and neuropsychiatric disorders associated with α7-nAChR dysfunction.

The α7 nicotinic acetylcholine receptor (α7-nAChR) is a ligand-gated ion channel expressed in neurons and nonneuronal cells of the human brain where it mostly forms homopentameric receptors composed of five α7 subunits (1, 2). This nAChR subtype is widely distributed in the central nervous system (CNS), although its expression is particularly prominent in the hippocampus and prefrontal cortex, two key regions involved in neurocognitive function (see Ref. (3) and references therein). The α7-nAChR presents a rapid desensitization and a high permeability to Ca^2+^ that exceeds that of NMDA receptors, implying that the former receptor can act as a precise modulator of the intracellular Ca^2+^ concentration [Ca^2+^]_i_ that triggers multiple responses in neurons (1, 4, 5, 6, 7). At the subcellular level, activation of presynaptic α7-nAChRs on nerve terminals in the hippocampus and other brain regions facilitates the exocytotic release of several neurotransmitters, including GABA, glutamate, dopamine, and noradrenaline, while in a postsynaptic location, these receptors activate multiple signaling cascades that promote neuronal plasticity and cell survival processes (8, 9, 10, 11, 12, 13, 14, 15, 16, 17, 18, 19, 20, 21, 22, 23, 24).

Given the determinant role of α7-nAChRs regulating the CNS synaptic transmission and plasticity that underlie the normal processes of attention, cognition, learning, and memory (See Ref. (25) and references therein), receptor dysfunction due to decreased expression and/or activity has been linked to a wide array of neurocognitive disorders, including Alzheimer's disease, schizophrenia, bipolar disorder, attention deficit hyperactivity, autism, and epilepsy (23, 26, 27, 28, 29). Hence, it is a huge challenge to identify endogenous or exogenous mechanisms that could alter α7-nAChR activity and thereby contribute to managing the abovementioned disorders.

Recently our group identified a possible endogenous candidate that may interfere with α7-nAChR function, the *CHRFAM7A* chimeric gene, which is evolutionarily a relatively recent gene since it appears in the human genome after its divergence from other higher primates (30). The new hybrid gene results from partial duplication (exons 5–10) of the parent *CHRNA7* gene, coding the α7 subunit that forms the α7-nAChR, fused to the *FAM7A* genetic element (31, 32). Although *CHRFAM7A* expression has been associated with neurocognitive disorders such as schizophrenia, psychosis, bipolar disorder, autism, and dementia (33, 34, 35), the functional role of the chimeric gene was long unidentified until we reported that its product, the dupα7 subunit, acted as a dominant negative regulator of α7-nAChR-induced currents in a pioneering electrophysiological study conducted in *Xenopus* oocytes (36). Our finding was corroborated shortly afterward by others, also in oocytes (37) and, a few years later, by our own group in diverse mammalian cell types. Thus, we reported that dupα7 overexpression inhibits, both *in vitro* and *in vivo*, α7-nAChR-mediated protumorigenic activity in human cell lines from non-small-cell lung cancer (NSCLC) (38). Furthermore, using GH4C1 rat pituitary cells and RAW264.7 mouse macrophages transfected with epitope- or fluorescent protein-tagged α7 or dupα7 constructs, we identified the mechanism underlying dupα7 interference in α7-nAChR function. This mechanism consists of the physical interaction between dupα7 and α7 subunits generating heteromeric nAChRs that largely remain mainly trapped in the endoplasmic reticulum (39). Thus, the dupα7 sequestration of α7 subunits reduced membrane expression of functional homomeric α7-nAChRs, attenuating their recognized anti-inflammatory capacity in lipopolysaccharide-stimulated macrophages (39).

The α7 subunit and its duplicate form are naturally coexpressed in the same human cell types, including neuronal, immune, or tumor cells (see Ref. (40) and references there). Thus, it is to be expected that the dupα7 subunit would behave as an endogenous negative regulator of α7-nAChR-mediated activity in neurons, just as it does in macrophages or tumor cells (38, 39). This last hypothesis, still unexplored, is the one addressed here using the SH-SY5Y human neuroblastoma cell line to assess: (i) the physical interaction between α7 and dupα7 subunits in fluorescent protein-tagged α7/dupα7 transfected cells; and (ii) the functional impact of the above interaction in cell variants with different dupα7 expression levels. Immunoblotting, quantitative real-time polymerase chain reaction (RT-PCR), Föster resonance energy transfer (FRET) analysis combined with functional assays of α7-nAChR-activity, in either single cells or cell populations of the above cell variants, allowed us to establish that the dupα7 subunit negatively regulates α7-nAChR-mediated control of exocytotic neurotransmitter release in neuronal cell types.

## Results

### Selection of clones with stable overexpression of dupα7

**Figure 1:**
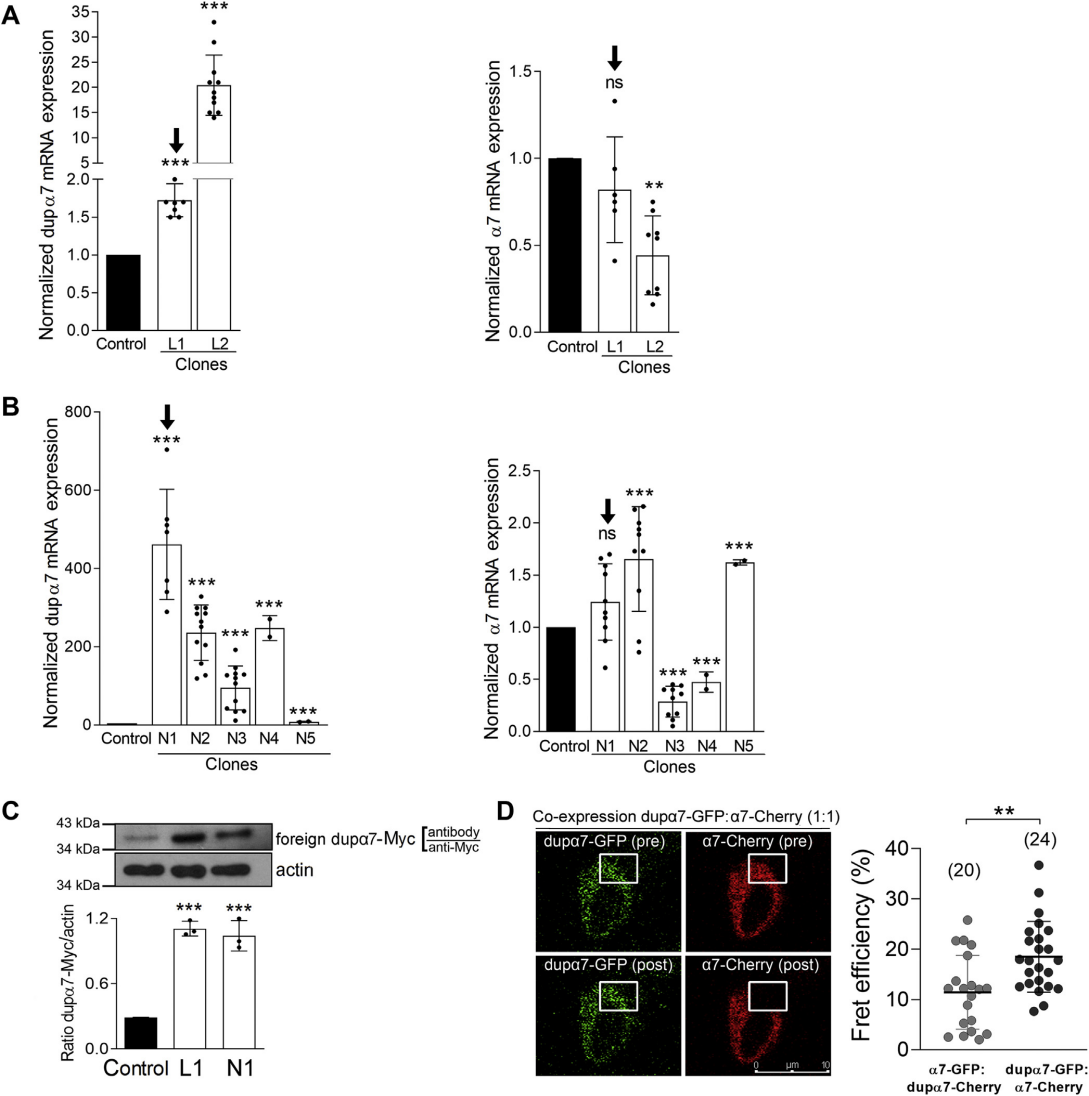
**Expression levels of dupα7 and endogenous α7 subunits in control SH-SY5Y cells or in cells with stable dupα7-myc overexpression (Clones).***A* and *B*, normalized expression of the mRNAs of both nicotinic subunits determined by qPCR in control cells (value = 1) and in the clones obtained after lipofection (L1 and L2) or nucleofection (N1–N5). The *solid circles* overlaid on the bar graphs represent individual data points obtained in independent cultures for each condition. The error bars show mean ± SD. ^∗^^∗^*p* < 0.01 and ^∗^^∗^^∗^*p* < 0.001 after comparison with the control. The two clones (L1 and N1) selected for the subsequent functional study are indicated by the *black arrow*. *C*, expression level of the foreign dupα7-myc protein, determined by immunoblot with the anti-myc antibody, in control cells or in the selected clones. At the *top*, a typical immunoblot; at the *bottom*, the protein expression values normalized with respect to β-actin expression. The error bars show mean ± SD from three independent cultures and solid circles the individual values for each condition. ^∗^^∗^^∗^*p* < 0.001 compared with control. *D*, physical interaction between α7 and dupα7 subunits in SH-SY5Y cells evaluated by FRET efficiency analysis. Cells were transfected with the construct pairs α7-GFP:dupα7-Cherry or dupα7-GFP:α7-Cherry, at a ratio of 1:1. On the *left*, representative confocal images of FRET analysis performed in the selected area of a cell cotransfected with dupα7-GFP:α7-Cherry, where the fluorescence emitted by dupα7-GFP in the framed area is shown before (pre) and after (post) α7-Cherry photobleaching; scale bar 10 μm. On the *right*, scatter plots (mean ± SD) representing individual data points of FRET efficiency values (expressed as a percentage) determined in the number of cells analyzed (in parentheses) from three independent cultures. ^∗^^∗^*p* < 0.01 after comparing the indicated values.

In order to avoid differences in the expression levels of dupα7 mRNA among different SH-SY5Y cultures transiently transfected with the dupα7.pcDNA3.1/Myc-His construct, we stably transfected the cells with this construct, or with its corresponding empty vector pcDNA3.1/Myc-His, as described in the corresponding Methods section. A total of seven positive clones resistant to G418 obtained by lipofection (L1 and L2) or nucleofection (N1-N5) of dupα7-Myc were initially selected. In all of them, the dupα7 or α7 mRNA levels were analyzed by qPCR in order to select those clones that, together with the clear overexpression of dupα7, presented endogenous α7 mRNA levels similar to those found in nontransfected cells (Control). We found two clones (L1 and N1) that fit the above requirements (Fig. 1, *panels* *A* and *B*). Since dupα7 mRNA overexpression is much more pronounced in the N1 than in the L1 clones, we proceeded to determine if this difference was maintained at the protein level. Immunoblot data using the anti-Myc antibody to detect the foreign dupα7-Myc protein overexpressed in L1 and N1 clones show that there were no significant differences in protein expression levels (Fig. 1*C*), so both clones were assayed in parallel throughout our study.

### Physical interaction of α7 and dupα7 subunits in SH-SY5Y cells

Using FRET confocal imaging analysis in SH-SY5Y cells cotransfected with two pairs of α7 and dupα7 constructs (α7-GFP:dupα7-Cherry or dupα7-GFP:α7-Cherry), we evaluated whether dupα7 could physically interact with α7 subunits, thus modulating the α7-nAChR-mediated control of neurotransmission. The results (Fig. 1*D*) provide evidence that both nAChR subunits were in sufficient proximity to interact with each other as part of a heteropentameric nAChR. Thus, the left panel of the figure shows representative confocal images acquired before (pre) and after (post) acceptor [α7-Cherry] photobleaching at 561 nm in the framed area; the increase in emission at 488 nm of the donor [dupα7-GFP (post)] in the cell analyzed after coexpression of the dupα7-GFP:α7-Cherry pair is worth noting. The right panel of the figure represents the scatter plots of individual data points corresponding to the FRET efficiency values, expressed as a percentage of the maximum efficiency, obtained in the region selected for acceptor photobleaching in cells transfected with the two pairs of constructs assayed in this study at a 1:1 ratio. The error bars represent mean ± standard deviation (SD). The significantly higher FRET efficiency values when dupα7 was the donor of the pair are also worth noting. Consequently, the subsequent experiments were designed to evaluate the functional consequences of this dupα7/α7 interaction, in both cell populations and single cells.

### Overexpression of dupα7 reduces the [Ca^2+^]_i_ signal evoked by α7-nAChRs in populations of SH-SY5Y cells

**Figure 2:**
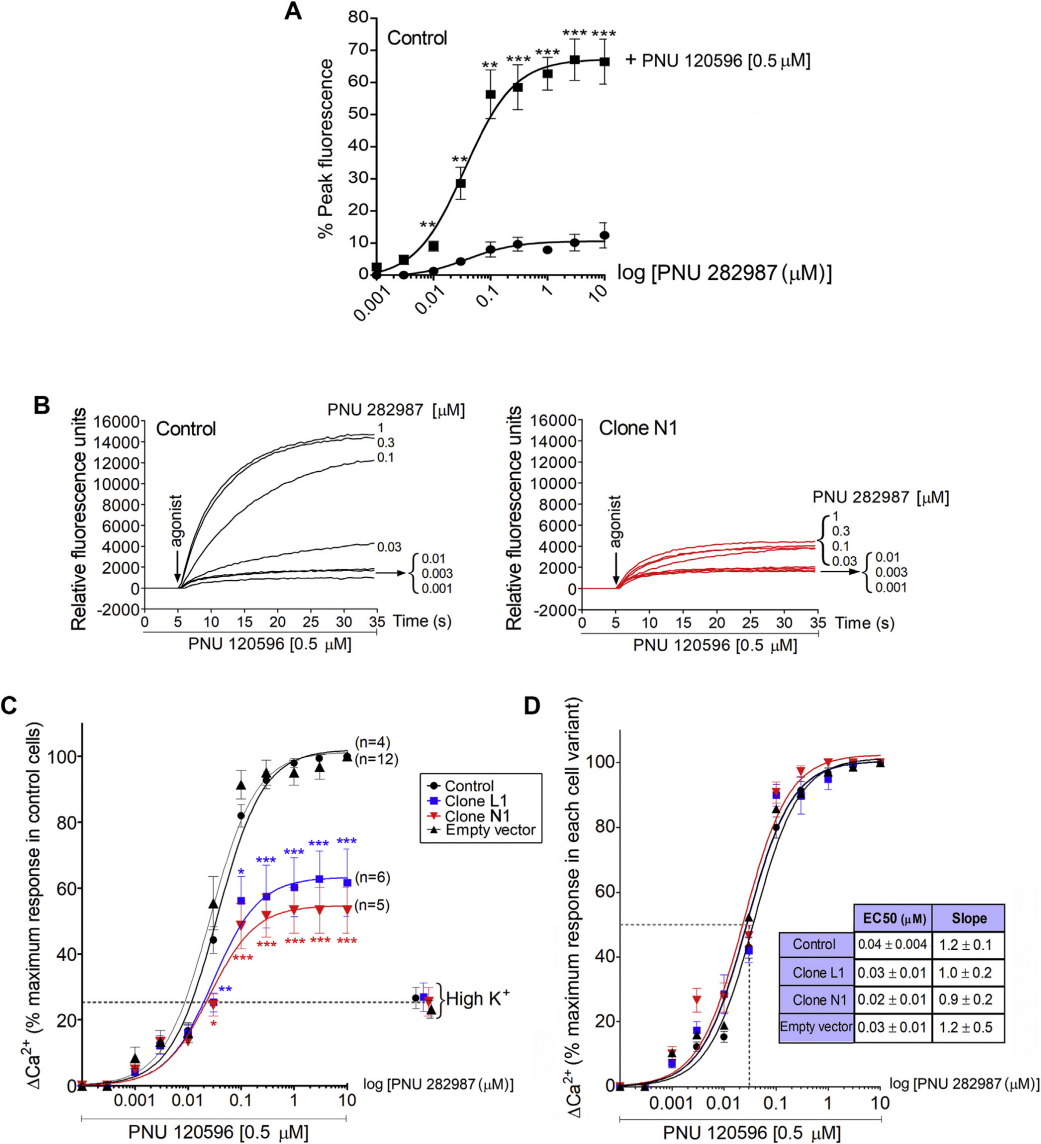
**Effect of dupα7 overexpression on the α7-nAChR-mediated [Ca**^**2+**^**]**_**i**_**signal in populations of SH-SY5Y cells.***A*, [Ca^2+^]_i_ signal induced by increasing concentrations of the selective α7-nAChR agonist, PNU 282987 (1 nM–10 μM), in the absence or presence of PNU 120596 (0.5 μM), a positive allosteric modulator (PAM) of the receptor. Peak fluorescence values induced by the agonist in both experimental conditions, obtained in triplicate, were expressed as a percentage of F_max_ − F_min_ (mean ± S.E.M.) and correspond to four independent cell cultures. ^∗^^∗^*p* < 0.01 and ^∗^^∗^^∗^*p* < 0.001 after comparing the same concentration of the agonist in both curves. *B*, original traces of fluorescence intensity induced by increasing concentrations of PNU 282987 (+PAM) in control cells (*black traces*; *left panel*) or in cells expanded from the N1 clone (*red traces*; *right panel*). The signal was monitored for 35 s and the PNU 282987 (agonist) was added after measuring basal fluorescence for 5 s, as is indicated by the *arrow*. *C* and *D*, pooled results of the normalized [Ca^2+^]_i_ responses (Δ[Ca^2+^]_i_) evoked by increasing concentrations of PNU 282987 (+PAM) in the four cell variants tested [control cells, clones L1 and N1 or transfected with empty vector]; finally, a depolarizing stimulus of high K^+^ (70 mM; 1 s) was applied at the end of the experiment. The Δ[Ca^2+^]_i_ signals were normalized as a percentage of the maximum response induced by PNU 282987 (3 μM; 100%) in control cells (panel *C*) or in the corresponding cell variant (panel *D*). Values are mean ± S.E.M. from the number of independent cultures indicated in parentheses for each cell variant in panel *C*. Table inserted (at the right of panel *D*) shows the EC_50_ and Slope values obtained from the concentration–response curves of PNU 282987 in the four cell variants tested.

One of the most distinctive functions of α7-nAChR in neurons is to promote the exocytotic release of several neurotransmitters as the result of the [Ca^2+^]_i_ rise induced by receptor stimulation. Therefore, the next experiments aimed to evaluate whether overexpression of dupα7 in SH-SY5Y cells interfered with the [Ca^2+^]_i_ response induced by 1-s pulses of increasing concentrations of PNU 282987 (1 nM up to 10 μM) applied to cell populations. To amplify the responses induced by the selective α7-nAChR agonist and thus facilitate the subsequent analysis of their possible modulation by dupα7, a fixed concentration of the positive allosteric modulator (PAM) of the α7-nAChR (PNU 120596; 0.5 μM) was added to the cell medium from 10 min before and during the α7-nAChR agonist pulse. Figure 2*A* shows the concentration–response curves to the agonist in nontransfected cells (Control), in both the absence and presence of the PAM; it can be observed that the last agent greatly enhanced the agonist-induced response at all tested concentrations. Figure 2*B* shows the original fluorescence traces induced by different concentrations of PNU 282987 (+PAM) in control cells (black traces; left panel) or in cells expanded from the N1 clone (red traces; right panel). Figure 2, *C* and *D* show pooled results from independent cultures (n = 4–12) of normalized [Ca^2+^]_i_ responses (Δ[Ca^2+^]_i_) evoked by increasing concentrations of PNU 282987 in the four cell variants tested [control cells, and cells overexpressing dupα7 (clones L1 and N1), or empty vector]. The Δ[Ca^2+^]_i_ signals were normalized as a percentage of the maximum response induced by PNU 282987 (3 μM; 100%) in control cells (Fig. 2*C*) or in the corresponding cell variant (Fig. 2*D*). The analysis of variance (ANOVA) applied to data in Figure 2*C* showed that while the Δ[Ca^2+^]_i_ signal induced by PNU 282987 in cells overexpressing the empty vector was indistinguishable from that found in control cells, the overexpression of dupα7 significantly reduced the α7-nAChR-mediated signal, particularly at the highest agonist testing concentrations (from 30 nM to 10 μM), but not the signal generated by a depolarizing stimulus of high K^+^ (70 mM, 1 s). The table inserted in Figure 2*D* shows the EC_50_ and slope values obtained from the concentration–response curves of PNU 282987 in the four cell variants tested; the application of the above statistical analysis to both parameters did not show significant differences between the Δ[Ca^2+^]_i_ signals generated in the four variants.

### Inhibition of the α7-nAChR-mediated [Ca^2+^]_i_ signal in single SH-SY5Y cells by dupα7 overexpression

**Figure 3:**
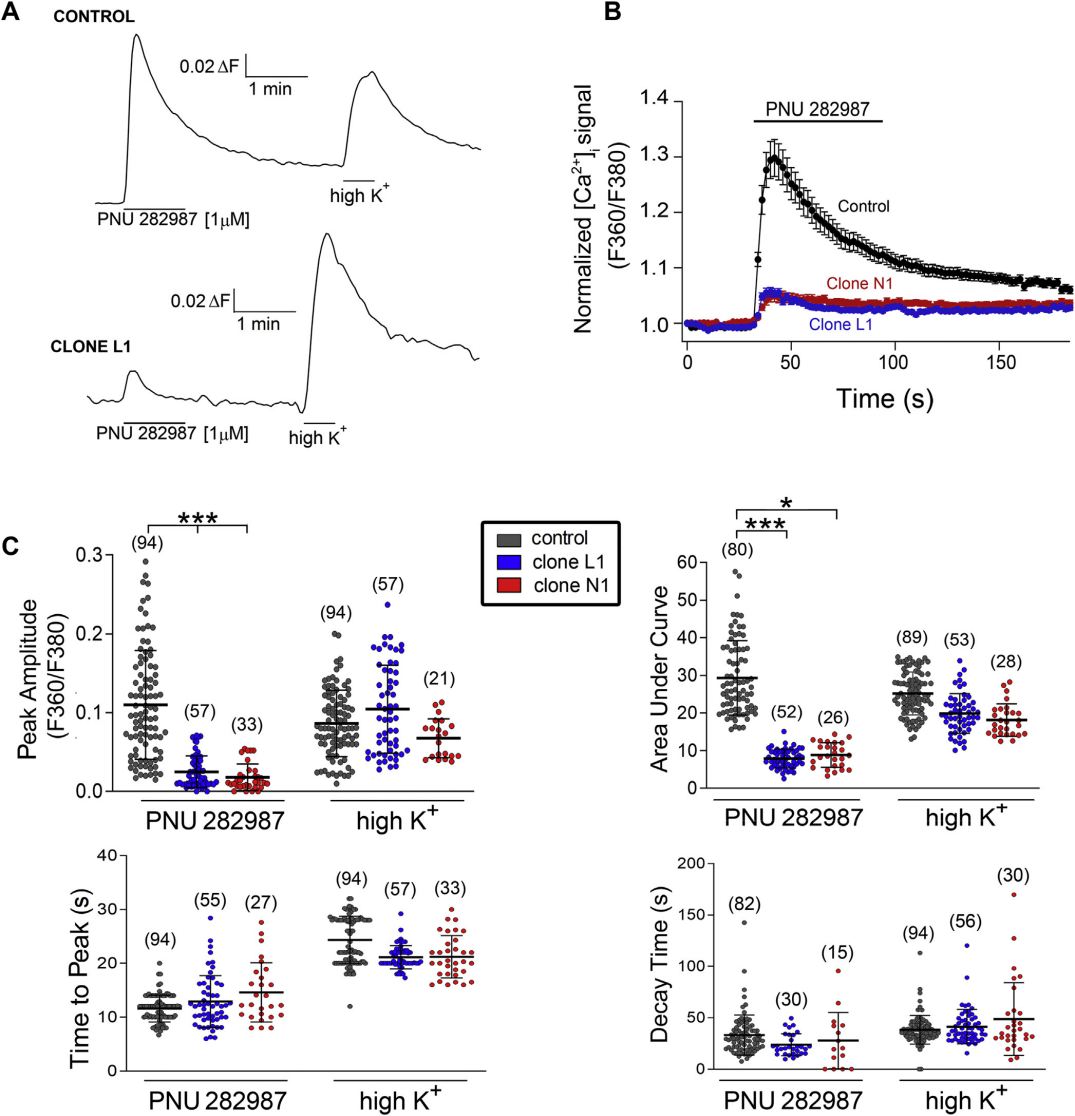
**Analysis of various kinetic parameters related to the [Ca**^**2+**^**]_i_ signal generated by PNU 282987 or high K^+^ in single control or dupα7-overexpressing SH-SY5Y cells.***A*, original traces of fluorescence signal (ΔF_Ratio_) induced by two successive pulses [1 μM PNU 282987 (+PAM, 1 μM) and 100 mM K^+^] applied 1 min apart to a Control cell or to a cell from Clone L1. *B*, time-course of the normalized [Ca^2+^]_i_ signal generated by the α7-nAChR agonist (+PAM) in individual cells from the three cell variants assayed (control and N1 and L1 clones). Each value represents mean ± S.E.M. of three cells for each variant. *C*, scatter plots of individual data points relative to different kinetic parameters obtained from the analysis of the [Ca^2+^]i signal evoked by PNU 282987 (+PAM) or high K^+^ in single cells corresponding to the three cell variants assayed. In parentheses, the number of cells from each variant. The error bars show mean ± SD. ^∗^*p* < 0.05 and ^∗^^∗^^∗^*p* < 0.001 after comparing the indicated values.

To gain further insights into dupα7 negative regulation of the α7-nAChR-induced [Ca^2+^]_i_ rise, we evaluated this regulation at the single cell level by fluorescence microscopy. The SH-SY5Y cells tested in these experiments belong to the next three cell variants: control, L1 clone, and N1 clone. Individual cells were stimulated with a 1-min pulse of PNU 282987 (1 μM) in the presence of PAM (1 μM) added to the perfusion medium 20 min before and during the application of the stimulus. After a washout period, the cells were exposed to a final high K^+^ pulse (100 mM, 30 s) to exclude from subsequent analyses those cells that had not responded to the depolarizing stimulus. Figure 3*A* shows original traces of the fluorescence increase (ΔF) induced by the above two pulses applied successively in two Fura 2-loaded cells representative of the Control (upper panel) and the L1 clone (lower panel) groups. Note that overexpression of dupα7 markedly reduces the α7-nAChR-mediated signal but not that induced by the depolarizing stimulus. In fact, the high K^+^-evoked response is higher in the dupα7 overexpressing cell than in the control cell, probably because the [Ca^2+^]_i_ signal induced by PNU 282987 in the latter cell had not yet returned to the basal level when the depolarizing stimulus was applied. Figure 3*B* shows pooled time-course results of the [Ca^2+^]_i_ signal evoked by PNU 282987 in 3 to 4 individual cells belonging to each tested cell variant; the signals recorded in the clones were normalized with respect to the signal obtained in control cells. Blockade of the α7-nAChR-induced signal was clearly seen in L1 and N1 clones with respect to control cells, but there were no significant differences in the blocked signal between the two types of clones. Figure 3*C* shows scatter plots of individual data points and statistical analyses of different kinetic parameters relative to the [Ca^2+^]_i_ signal induced by PNU 282987 or high K^+^ in single cells from the three variants (control, clone L1, and clone N1) from independent cultures. The error bars represent mean ± SD of the values obtained in the number of cells appearing in parentheses. The overexpression of dupα7 significantly reduced the “Peak Amplitude” and the “Area Under the Curve” of the [Ca^2+^]_i_ signal induced by α7-nAChR but not the signal evoked by the depolarizing stimulus. None of the other analyzed kinetic parameters, independently of the stimulus, was significantly affected by dupα7 overexpression.

### Overexpression of dupα7 reduces the α7-nAChR-mediated exocytotic response in single SH-SY5Y cells

**Figure 4:**
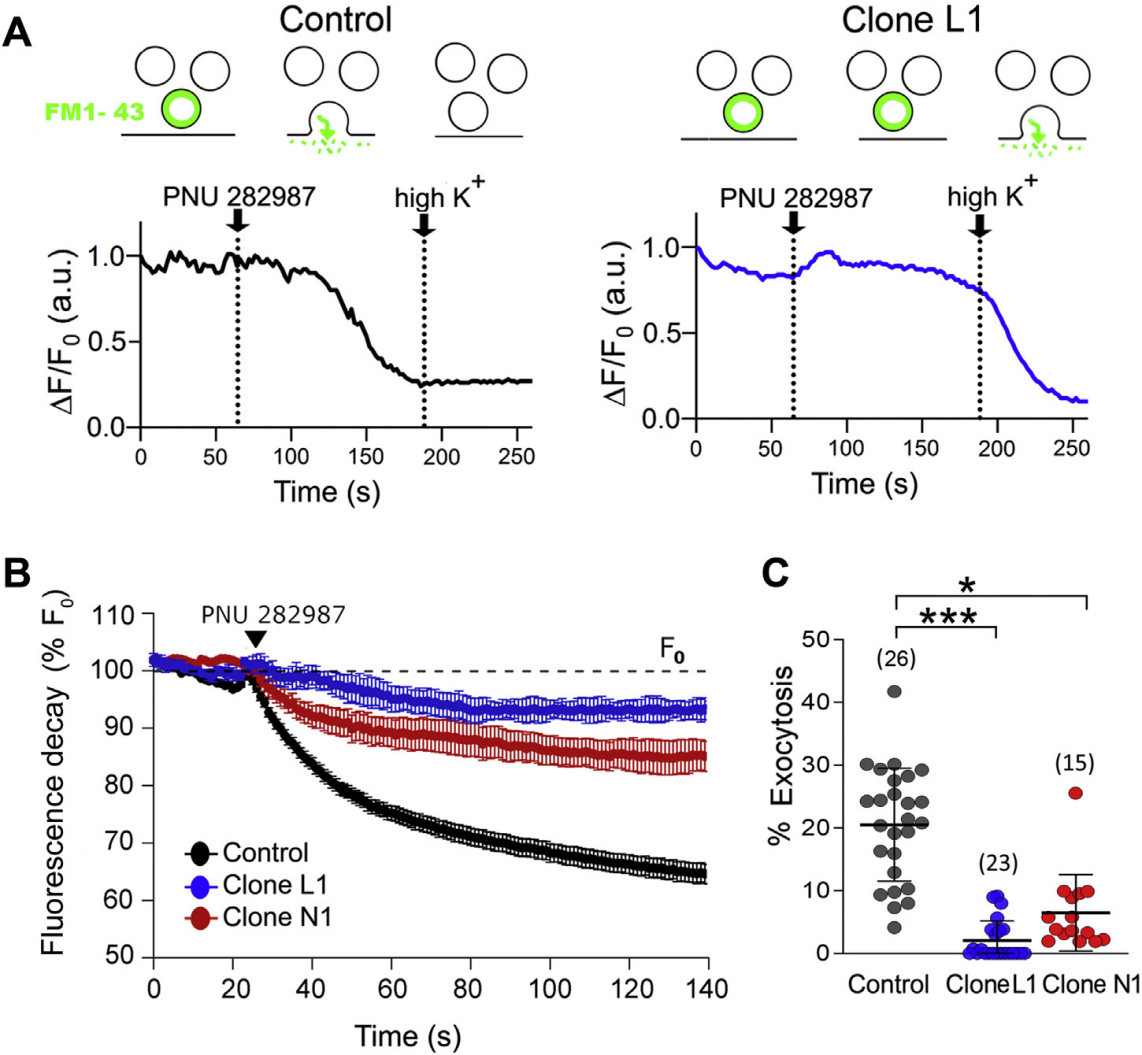
**Effect of dupα7 overexpression on the exocytotic responses elicited by PNU 282987 and high K^+^ in single control or dupα7 overexpressing SH-SY5Y cells.** Synaptic vesicles were loaded with the fluorescent FM1-43 membrane dye as described in the Method section. The cells were then thoroughly washed and subsequently subjected to two successive stimulations with 1 μM PNU 282987 (+PAM, 1 μM) and 100 mM K^+^ to induce fusion of labeled vesicles with the plasma membrane and subsequent exocytotic release of FM1-43, which is deduced by the loss of the fluorescent signal of the probe (destaining) in the aqueous solution. *A*, original traces of the fluorescent signal in response to both stimuli applied to a Control cell (*left*) or to a cell overexpressing dupα7 (Clone L1; *right*). In the *upper part*, diagram representing the FM1-43 dye used to evaluate the exocytotic responses to different stimuli in individual cells. *B*, timecourse of the fluorescent signal decay (destaining) reflecting the PNU 282987-induced exocytotic response in control cells or in cells from L1 and N1 clones; values represent mean ± S.E.M. from several cells (n = 3–4) for each cell variant. F_0_ corresponds to the maximum fluorescence (100%) before the addition of the stimulus (*black arrow*). *C*, scatter plots of individual data points reflecting the percentage of the exocytotic response induced by PNU 28297 in single cells from the three cell variants; error bars show mean ± SD of the number of cells analyzed for each variant (in parentheses). ^∗^*p* < 0.05 and ^∗^^∗^^∗^*p* < 0.001 compared with control cells.

The [Ca^2+^]_i_ rise induced by α7-nAChRs in neurons activates the SNARE protein complex and the consequent discharge of the vesicular content (neurotransmitter) into the synaptic space by an exocytotic mechanism. Thus, it is likely that the α7-nAChR-mediated exocytotic response in SH-SY5Y cells is affected by dupα7 overexpression, as is the [Ca^2+^]_i_ signal induced by the same receptor subtype. The following experiments were aimed at evaluating this hypothesis at the single cell level. For this, synaptic vesicles of SH-SY5Y cells belonging to the three cell variants tested in this study (control, clone L1, and clone N1) were loaded with the fluorescent FM1-43 dye as described in the corresponding Methods section. Subsequently, the exocytosis of the labeled vesicles was promoted by two successive 1-min pulses of 1 μM PNU 282987 (+PAM, 1 μM) and high K^+^ (100 mM), separated by a washing period. Figure 4*A* shows two original destaining traces (loss of the fluorescent signal) indicative of the extent of the exocytotic response induced by the two stimuli in a Control cell and in a cell overexpressing dupα7 (Clone L1). The upper diagram of both registers illustrates the two key steps of what happened with the labeled vesicles in each cell type in response to the two stimuli: 1) PNU 282987 induced vesicular exocytosis (drop in the fluorescent signal) in the Control cell but not in the one with dupα7 overexpression; and 2), in contrast, high K^+^ was not able to promote exocytosis of the labeled vesicles in the Control cell (due to its depletion), whereas it did in the L1 cell. Figure 4*B* shows the timecourse for fluorescence decay in response to PNU 282987 in several cells from each cell variant: Control (n = 3), L1 (n = 3), and N1 (n = 4). Data are expressed as a percentage of the maximum fluorescence (F_0_, considered as 100%) recorded before the application of the stimulus and they represent mean ± S.E.M. of the values obtained in each cell variant. Figure 4*C* shows the scatter plots of individual data points reflecting the extent of exocytosis induced by PNU 282987 in single cells from the above cell variants. The values correspond to the maximum drop of fluorescence, expressed as a percentage of F_0_, induced by the nicotinic agonist once the timecourse of the fluorescence decay had stabilized. The error bars represent mean ± SD of the number of cells indicated in parentheses. The statistical analysis of the above results indicates that dupα7 overexpression significantly reduced the exocytotic response induced by α7-nAChR in single SH-SY5Y cells, as was expected to occur based on the dupα7 effects on the [Ca^2+^]_i_ signal triggered by this receptor subtype in these cells (Figs. 2 and 3).

### Silencing dupα7 gene expression with siRNAs positively modulates α7-nAChR-induced DA release in populations of SH-SY5Y cells

**Figure 5:**
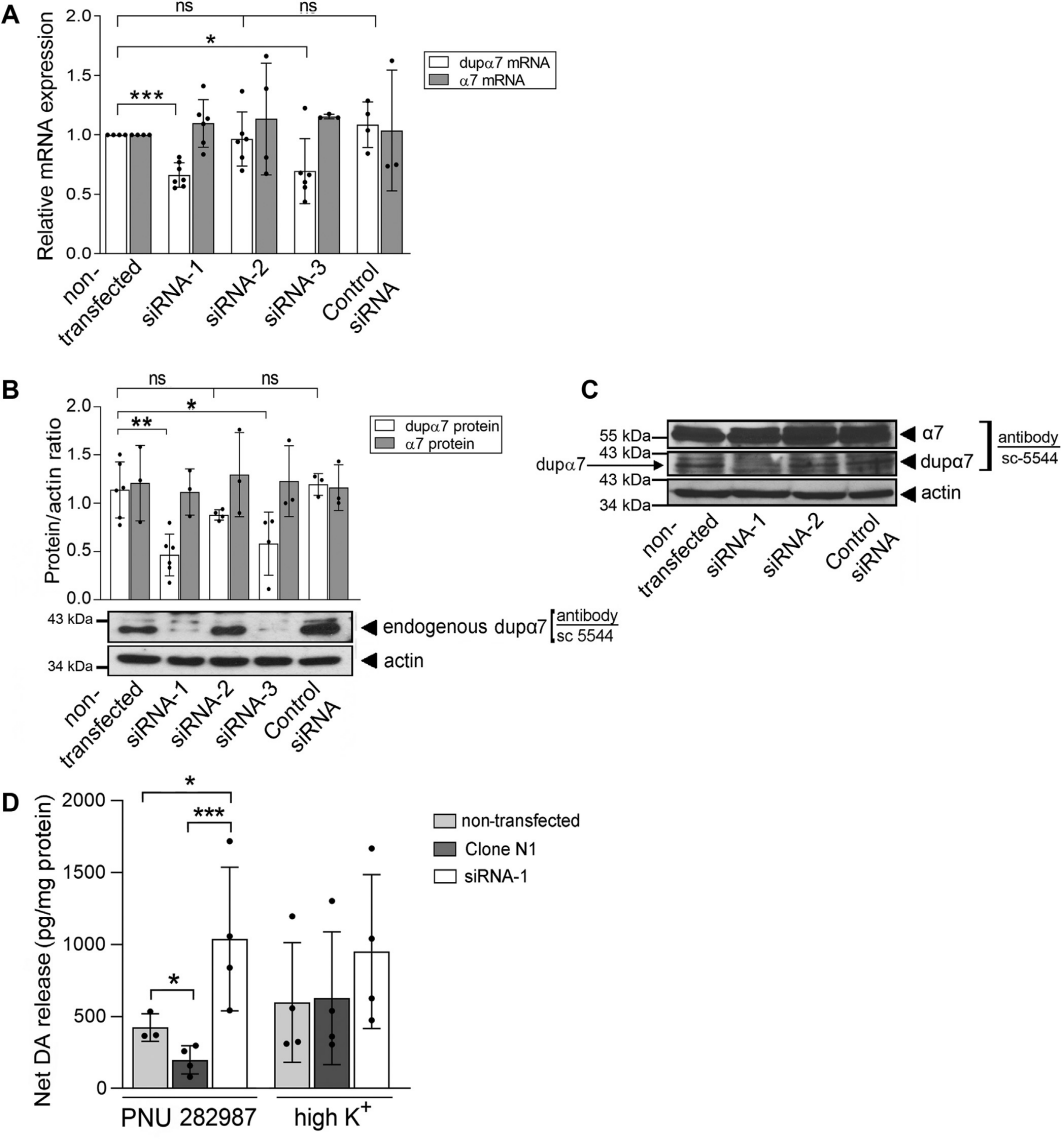
**Silencing effectiveness of different siRNAs on *dupα7* gene expression and its functional consequence on α7-nAChR-mediated neurotransmitter release in populations of SH-SY5Y cells.** The efficacy of three different siRNAs designed for selectively silencing *CHRFAM7A* gene expression was double-checked by qPCR and immunoblot. *A*, bar graph showing mean ± SD of the normalized expression of dupα7 and α7 mRNAs in cells transfected with the tested siRNAs (1, 2 or 3); cells transfected with a commercial siRNA (control siRNA) and nontransfected cells were used as negative and positive controls, respectively. *B*, bar graph representing mean ± SD of the dupα7/β-actin or α7/β-actin protein ratio determined in the same population of cells assayed above; at the *bottom*, one original blot of a membrane cut above the 43 kDa molecular marker and incubated with the sc-5544 antibody to exclusively view the endogenous dupα7 subunit (≈41 kDa). *Solid circles* overlaid on the bar graphs in panels *A* and *B* represent individual data points obtained in independent cultures for each condition. ^∗^*p* <0.05, ^∗^^∗^*p* <0.01 and ^∗^^∗^^∗^*p* <0.001 compared with non-transfected cells. *C*, original blot of a single membrane incubated with the sc-5544 antibody that recognizes two bands whose sizes correspond to the α7 (≈57 kDa) and dupα7 proteins. Only siRNA-1 and siRNA-3 showed selectivity and effectiveness in silencing dupα7 mRNA and protein expression, leaving the parent *CHRNA7* gene expression unchanged. *D*, exocytotic dopamine (DA) release induced by 1 μM PNU 282987 (+PAM, 0.5 μM) or high K^+^ (100 mM) in populations belonging to three cell types: nontransfected, with stable dupα7 overexpression (clone N1), and with endogenous dupα7 silencing with siRNA-1. Net DA release, determined by ELISA, was normalized to the cell protein content. *Solid circles* overlaid on the bar graph represent individual data points obtained in 3 to 4 independent cultures for each condition. The error bars show mean ± SD. ^∗^*p* < 0.05 and ^∗^^∗^^∗^*p* < 0.001 after comparing the indicated values.

Once it was verified that overexpression of foreign dupα7 subunits negatively regulates α7-nAChR-mediated exocytotic response in single SH-SY5Y cells (Fig. 4), we sought to explore whether endogenous dupα7 subunits expressed in this human neuronal cell model actually have a functional role in the control of α7-nAChR-mediated neurotransmission. To explore this possibility, we silenced *dupα7* gene expression with three specific siRNAs, using a commercial siRNA (control siRNA) as negative control. The silencing effectiveness of each siRNA was double-checked by RT-PCR and immunoblot. The results of both analyses revealed that while siRNA-2 was ineffective in silencing gene expression, siRNA-1 and siRNA-3 significantly reduced it, both at the mRNA (Fig. 5*A*) and at the protein (Fig. 5, *panels* *B* and *C*) levels. Figure 5 (*panels* *A*–*C*) also confirms the specificity of the last two siRNAs in silencing *dupα7* gene expression without affecting the expression of the parent *α7* gene, despite the high homology between the nucleotide sequences of both genes. The original immunoblot (Fig. 5*C*) shows that the sc-5544 antibody recognized two bands of different sizes in the same membrane that correspond to the α7 (≈57 kDa) and dupα7 (≈41 kDa) subunits. Note that the heavy expression of α7 makes it difficult to see dupα7 expression in the same membrane. To overcome this drawback and better visualize the band corresponding to the duplicated subunit, the membrane of the immunoblot was cut above the 43 kDa molecular weight marker before being incubated with the antibody (Fig. 5*B*, *bottom*). Since siRNA-1 was slightly more efficient than siRNA-3 in silencing *dupα7* gene expression, the former was chosen to evaluate the functional role of endogenous dupα7 in regulating α7-nAChR-mediated exocytotic dopamine (DA) release in populations of SH-SY5Y cells. To carry out these experiments, neurotransmitter release in response to 1 μM PNU 282987 (+PAM, 0.5 μM) or high K^+^ (100 mM) was determined by ELISA in the following three cell variants: 1) nontransfected; 2) with stable overexpression of foreign dupα7 protein (clone N1); or 3) with silenced endogenous expression of dupα7 (siRNA-1). Figure 5*D* shows the bar graph and the error bars (mean ± SD) of the net DA release induced by cell incubation (3.5 min) with one or the other stimulus for each experimental condition assayed in three to four independent cell cultures. The solid circles overlaid with the bar graphs represent individual data points obtained for each condition. Compared with nontransfected cells, the silencing of dupα7 expression significantly increased (by more than twice) the α7-nAChR-induced secretory response, while overexpression of the duplicated subunit significantly reduced that response, as we had previously observed with the exocytotic process in single cells (Fig. 4). In contrast, the secretory response induced by the depolarizing stimulus remained unchanged whatever the level of dupα7 expression in the cells (Fig. 5*D*).

## Discussion

The α7-nAChR is highly expressed in the human brain, where it exercises essential and well-defined functions that include, among many others, the control of cellular Ca^2+^ signaling and synaptic transmission. The present study characterizes, for the first time in a neuronal cell model, the negative regulation of these important receptor-mediated functions by the human-specific duplicated α7 form, dupα7. Our data, in single cells and in cell populations, reveal that both the [Ca^2+^]_i_ signal and the exocytotic response generated by selective stimulation of α7-nAChRs were either significantly inhibited by stable overexpression of dupα7 or augmented after silencing *dupα7* gene expression with specific siRNAs.

Listed below are the reasons that respectively motivated the selection of SH-SY5Y cells and the dupα7.pcDNA3.1/Myc-His construct as the neuronal model and tool to identify positive clones with stable dupα7 overexpression in our study: 1) the cell line endogenously expresses functional α7-nAChRs whose activation promotes an exocytotic dopamine release that mimics what occurs in the cortical, hippocampal, or striatal neurons (17, 20, 41, 42); 2) as human cells, SH-SY5Y naturally express dupα7 subunits; 3) the construct contains the –Myc– epitope that allows it to pick up positive clones overexpressing dupα7 since, otherwise, the high homology in the peptide sequences between the dupα7 and α7 subunits causes commercially available antibodies to cross-react with both proteins preventing immunocytochemical identification of dupα7 overexpressing clones; 4) the small size of the –Myc– epitope (10 aa) used to recognize the dupα7 subunit would not interfere with heterometic α7/dupα7-nAChR activity, differently from the bulky Cherry or GFP fluorescent proteins.

Our first data reveal that transfection efficiency (number of positive clones) of the above construct in SH-SY5Y cells is higher after nucleofection than lipofection (data not shown), as also reported in other mammalian cell lines (43, 44, 45). Moreover, the dupα7 mRNA expression level was significantly higher in selected positive clones obtained by construct nucleofection (clones N1–N5) than in those obtained by lipofection (clones L1 and L2) (Fig. 1, *A* and *B*; *left* *panels*). This last finding could be due to plasmid distribution within the cell differing in accordance with the transfection method employed. Thus, the concentration of the plasmid in the cytoplasm of lipofected cells would be higher than in nucleofected ones due to a lower integration of the episome into the cellular chromosomal DNA (46).

In contrast to what would be expected from the above mRNA data, no significant differences were found in the dupα7-Myc protein expression levels between the two clone types, L1 and N1, obtained by lipofection or nucleofection (Fig. 1*C*). Although a general dogma in molecular biology is the existence of a direct correlation between mRNA and protein levels, this correlation is not found for certain messenger/protein pairs (47, 48). This lack of correlation could be due to the fact that translation of mRNA into protein is conditioned by multiple factors, such as the density of the ribosomes in the cell, the protein synthesis rates, or the existence of mRNA levels that exceed the capacity of cell translation because part of the mRNA occupies the polysomes (“ribosomal occupation”), thereby preventing the efficient translation of the remaining mRNA (49, 50, 51, 52).

FRET efficiency values (Fig. 1*D*) in the cells transfected with the two pairs of fluorescent protein-tagged α7 and dupα7 constructs used here (α7-GFP:dupα7-Cherry and dupα7-GFP:α7-Cherry) reveal that both nicotinic subunits are able to interact physically with each other, probably forming heteropentameric nAChRs, as we had previously reported in other mammalian cell types, such as GH4C1 or RAW264.7 cells (39). However, it should be noted that FRET efficiency values differ substantially among the three mammaliam cell types so far assayed in our laboratory, with RAW264.7 showing the highest values (39) and SH-SY5Y the lowest (present study). This finding is probably a consequence of a native expression of dupα7 subunits in SH-SY5Y cells but not in RAW264.7 or GH4C1 cells. Therefore, unlike the last two cell types, dupα7 subunits naturally expressed in SH-SY5Y cells could interfere with the assembly process of foreign fluorescent protein-tagged dupα7 and α7 subunits expressed in the cell, the latter being the subunits actually detected in the FRET analysis. Thus, the higher the endogenous expression of dupα7, the lower the FRET value reached in the cell. It is also interesting to note that when SH-SY5Y cells were transfected with the two pairs of constructs used in our study, the FRET efficiency values were significantly higher when the energy donor molecule (GFP) of the couple was dupα7 (Fig. 1*D*, scatter plots). This last finding agrees with our own previous data in RAW264.7 cells (39) as well as with data from another group in mouse neuroblastoma Neuro2A cells (53). The authors of the latter study justified their results by the low translation rate of dupα7 mRNA into its corresponding protein compared with that of α7 mRNA. Thus, dupα7-GFP subunits (acting as a donor) were more likely to lie adjacent to α7-Cherry subunits in the heteromeric nAChR than the alternative, that is, dupα7-Cherry subunits would be found in the vicinity of α7-GFP subunits forming the heteromeric nAChR.

Based on the above data as well as on previous findings by our group and others in *Xenopus* oocytes and in several mammalian cell lines, such as neuroendocrine GH4C1, immune RAW264.7, BOSC-23 kidney, or human NSCLC cells (36, 37, 39, 54, 55), we propose that dupα7 subunits could be colocated with full-length α7 subunits in SH-SY5Y cells forming heteromeric dupα7/α7-nAChRs to the detriment of fully functional homomeric α7-nAChR expression. If our proposal is true, then overexpression of dupα7 in SH-SY5Y cells would result in a decreased cellular responsiveness to α7-nAChR stimulation. The functional data produced by selective stimulation of α7-nAChRs in four variants of SH-SY5Y cells (control, empty-vector transfected cells, or clones L1 and N1) corroborates the above hypothesis and will be dicussed below.

Clones L1 and N1 are the ones selected here for functional studies as they show a similar extent of dupα7 protein overexpression (Fig. 1*C*) without modification of the endogenous α7 mRNA levels of control cells (Fig. 1, *A* and *B*; right *panels*). The results obtained in cell populations from both clones reveal that dupα7 overexpression significantly reduces the [Ca^2+^]_i_ signal generated by selective activation of α7-nAChR with the agonist PNU 282987 compared with signals in control or empty-vector transfected cells (56) (Fig. 2, *B* and *C*). As expected from previous studies (57, 58), the addition of the PAM-type II agent (PNU 120596) amplified the agonist-induced signal, as is clearly verifiable in control cells (Fig. 2*A*). Interestingly, the above dupα7 effect in cell populations was reproduced at the single-cell level, as evidenced by the significant reduction of the “Peak Amplitude” and “Area Under the Curve” of the [Ca^2+^]_i_ signal generated by selective stimulation of α7-nAChRs in individual cells from clones L1 or N1 compared with control cells (Fig. 3). The inability of PNU 282987 to induce a vigorous [Ca^2+^]_i_ signal in the two selected L1 and N1 clones, when compared with that achieved in control or empty vector-transfected cells, was not due to: 1) a deterioration in cell responsiveness since the four cell variants used in our study produce a similar [Ca^2+^]_i_ rise in response to a depolarizing stimulus, such as high K^+^, both in cell populations and in individual cells (Figs. 2*C* and 3); or 2) a decrease in the affinity of PNU 282987 for the expressed nAChR given the similarity of the EC_50_ values for the agonist found in the four evaluated cell variants (Fig. 2*D*, inserted table). Therefore, the above loss of effectiveness of PNU 282987 in clones L1 and N1 would be better explained on the basis of a reduction in the number of functional α7-nAChRs expressed on the cell surface in these latter cell variants as a result of dupα7 overexpression. Our interpretation was supported by previous studies in oocytes, RAW264.7, or PC12 cells that show that dupα7 overexpression significantly reduces the number of α-Bgtx binding sites on the cell membrane (36, 39, 55).

It is well known that the [Ca^2+^]_i_ rise induced by activation of α7-nAChRs in SH-SY5Y cells leads to exocytotic neurotransmitter (DA) release. Therefore, it is foreseeable that dupα7 overexpression in these cells would also cause a significant reduction in release of that transmitter. Our results in single cells from L1 or N1 clones indicate that this is the case (Fig. 4). Even more interesting, if possible, is the finding that dupα7 subunits naturally expressed by SH-SY5Y cells are capable of controlling the extent of exocytotic activity mediated by native α7-nAChRs, as deduced from the potentiation of the receptor-induced DA release in cell populations (Fig. 5*D*) after dupα7 gene silencing with specific siRNAs (Fig. 5, *A*–*C*). This latter finding suggests the presence of a “α7-nAChR tone” in CNS neurons that, maintained by naturally expressed dupα7 subunits, would regulate physiological synaptic transmission. This balanced crosstalk between dupα7 and α7-nAChRs could be lost when changes in the expression of the duplicate subunit secondary to certain pathological processes occur. It is also interesting to note that *dupα7* gene silencing with siRNA-1 enhances the α7-nAChR-mediated transmitter release only, but not that induced by cell depolarization with high K^+^, supporting the idea that the duplicated subunit acts by selectively interfering with ancestral receptor activity but not with any other kind of cell stimulus.

In summary, our study reveals that the dupα7 subunit behaves as a negative endogenous modulator of the exocytotic transmitter release controlled by α7-nAChRs in a human neuronal cell model, as we have previously demonstrated with other α7-nAChR-mediated functions in immune or tumoral cells (38, 39). Impaired expression and function of α7-nAChRs have been implicated in the pathogenesis of many neurological and psychiatric disorders (see Ref. (23) and references therein). In fact, other studies have established an association between the *CHRFAM7A* gene and certain neurocognitive diseases. Therefore, we propose that an upregulated expression of the latter gene, triggered by a yet unknown mechanism, could contribute to some of the above disorders mediated by deficient α7-nAChR activity in the CNS. This last proposal is strongly supported by the Kunni and colleagues study reporting an altered ratio of *CHRFAM7A*/*CHRNA7* transcripts (mainly due to overexpression of dupα7 mRNA) in postmortem dorsolateral prefrontal cortex of subjects with schizophrenia and bipolar disorder compared with control nonpsychiatric group (34).

## Experimental procedures

### Cell culture and reagents

Human neuroblastoma SH-SY5Y cell line was purchased from American Type Culture Collection ATCC. Cells were maintained in a Dulbecco's Modified Eagle Medium-F12 (DMEM-F12) from GIBCO (ThermoFisher Scientific) supplemented with 10% fetal calf serum, glutamax (1×), penicillin G sodium (100 units/ml), and streptomycin sulfate (100 μg/ml) in a 5% CO_2_ humidified incubator at 37 °C. Primary mouse anti-Myc and goat anti-β-actin monoclonal antibodies were purchased from Roche and Sigma-Aldrich, respectively. Primary rabbit polyclonal anti-α7/anti-dupα7 antibody (sc-5544) and secondary (HRP)-conjugated anti-mouse IgG and anti-goat IgG antibodies were obtained from Santa Cruz Biotechnology, while the secondary (HRP)-conjugated anti-rabbit IgG antibody was purchased from Bio-Rad. The siRNAs (1 and 3) were designed by one of the study authors (A. Chávez-Reyes) and synthesized by Ambion (ThermoFisher Scientific), while siRNA-2 and “siRNA negative control” were from Qiagen. PNU 282987 and PNU 120596 were from Abcam and Tocris Bioscience, respectively. Dopamine and geneticin (G418) were from Sigma-Aldrich. Dopamine High Sensitive ELISA kit was from DLD Diagnostika GMBH. The Lipofectamine 2000 and Nucleofection kits were from Invitrogen and Amaxa Biosystems, Lonza, respectively. The acetoxy-methyl ester form (AM) of the fluorescent Ca^2+^ indicators Fluo-4 and Fluo-2 and the FM1-43 membrane probe were from Molecular Probes, Invitrogen.

### SH-SY5Y cell transfection and selection of stable overexpressing dupα7-Myc transfectants

The dupα7.pcDNA3.1/Myc-His construct used for transfection was prepared in our laboratory as described elsewhere (39); it contains the full-length human dupα7 cDNA sequence in frame with the Myc-His tag. SH-SY5Y cells were transfected with the above plasmid or the corresponding empty vector (pcDNA3.1/Myc-His) using either the Lipofectamine 2000 or the Nucleofection kits according to the manufacturer's instructions as detailed elsewhere (39). Twenty-four hours after transfection, the cells were trypsinized and replated in culture medium supplemented with the aminoglycoside antibiotic G418 (800 μg/ml) used to select antibiotic-resistant stably transfected clones; single positive clones were picked up using cloning cylinders. Messenger and protein expression for dupα7 or α7 in expanded cells derived from each putatively positive clone were respectively analyzed by qPCR or immunoblot as detailed below.

### qPCR assay of mRNA expression in SH-SY5Y cells

Techniques for RNA extraction from cells and α7 or dupα7 mRNA expression analysis by qPCR from reverse-transcribed RNA using the SYBR green-based assays (Bio-Rad) and the ABI Prism 7500 Sequence Detector (Applied Biosystems) have been described elsewhere (39, 59, 60). Briefly, total RNA was extracted from SH-SY5Y cells with the RNeasy Mini kit (Qiagen,) and reverse-transcribed into cDNA using Taqman Reverse Transcription Reagents kit (Life Technology, Thermofisher Inc) according to the manufacturer’s instructions. The PCR amplification was performed using the following cycling conditions: 95 °C for 10 min, followed by 40 cycles at 95 °C for 15 s and 60 °C for 60 s. The primers used for PCR amplification of the corresponding transcript were: dupα7, forward 5’- CAATTGCTAATCCAGCATTTGTGG-3’ and reverse 5’-CCCAGAAGAATTCACCAACACG-3’; and α7, forward 5’-GCTGCAAATGTCTTGGACAGA-3’, and reverse 5’-AACAGTCTTCACCCCTGGATAT-3’. Analysis of the melting curves demonstrated that each pair of primers amplified a single product. The relative mRNA level of target transcripts was normalized with respect to two reference genes, beta-2 microglobulin (*B2M*) and ubiquitin C (*UBC*), using the 2^−ΔΔCt^ method and the SDS 2.0.6 software (Sequence Detection System, Applied Biosystems) as described elsewhere (59, 60). Primers used for PCR amplification of reference genes were: *B2M*, forward 5’-TGCCTGCCGTGTGAACCATGT-3’ and reverse 5’-TGCGGCATCTTCAAACCTCCTCCATGA-3’; *UBC*, forward 5’-GTTCCGTCGCAGCCGGGATT-3’, and reverse 5’-TGCATTGTCAAGTGACGATCACAGC-3’. Student’s *t*-test was used for statistical analysis of the data.

### Western blotting

SH-SY5Y cells were lysed, and the concentration of proteins in the cell lysates was determined with the BCA assay kit (Pierce BCA Protein Assay kit, Thermo Fisher Inc). Proteins were resolved by denaturing 7% SDS/PAGE gel electrophoresis, transferred to a PVDF membrane (Millipore Corporation), and immunoblotted with the appropriate antibodies as described elsewhere (36, 39, 61). The dilutions and incubation periods of the primary antibodies used to detect foreign and endogenous dupα7 subunits expressed in the cells were the anti-Myc (1:200; 2 h) and the sc5544 (1:500; 2 h), respectively. Although the last antibody recognizes both the dupα7 and α7 subunits, the first protein is perfectly distinguished from the second due to its smaller size (41 kDa *versus* 57 kDa). The secondary (HRP)-conjugated antibodies, anti-mouse IgG (1:5000) or anti-rabbit IgG (1:4000), were incubated at room temperature for 1 h. The resulting bands corresponding to dupα7 expression were detected using ECL Plus reagents (Amersham, GE Healthcare) and quantified by densitometry using the Image J software (National Institutes of Health, USA) and β-actin as the reference protein. Student's *t*-test was used for statistical analysis of these data.

### FRET analysis

The GFP- and pmCherry-N1-tagged constructs for both nAChR subunits (α7-pGFP, α7-pmCherry-N1, dupα7-pGFP, and dupα7-pmCherry-N1) were prepared in our laboratory by PCR using the dupα7.pSP64T and α7.pSP64T plasmids as templates and the set of primers described elsewhere (39). SH-SY5Y cells grown on glass coverslips treated with poly-L-lysine were nucleofected with the following two pairs of constructs (α7-GFP:dupα7-Cherry or dupα7-GFP:α7-Cherry), at a 1:1 ratio. Forty-eight hours after transfection, cells were fixed, rinsed, and mounted using citifluor AF-2. Images of selected regions in each cell were taken to determine FRET efficiency using the acceptor photobleaching technique and the Leica TCS SP5 Spectral confocal microscope as described elsewhere (39). Efficiency was calculated using the equation: FRET efficiency = 1 − (D_pre_/D_post_); where D_pre_ and D_post_ represent the fluorescence emitted by the donor (GFP) before and after photobleaching of the acceptor (Cherry) using the 561 laser. Student’s *t*-test was applied for statistical analysis.

### Measurement of [Ca^2+^]_i_ in SH-SY5Y cell populations using a microplate reader

Cells were plated in transparent bottom 96-well black plates (5 × 10^4^ cells/well) and incubated during 48 h at 37 °C. Afterward, cells were loaded with Fluo-4 AM (4 μM) and pluronic acid (0.02%) in serum-free culture medium for 45 min at 37 °C in the dark. Then cells were washed twice with Krebs-HEPES solution (in mM) [140 NaCl, 5.6 KCl, 1.2 MgCl_2_, 0.4 CaCl_2_, 10 HEPES, 11 d-glucose, pH 7.4] and kept at room temperature for 15 min before beginning the experiment. To induce the [Ca^2+^]_i_ rise, the cells were stimulated with increasing concentrations of a selective α7-nAChR agonist, PNU 282987. Whenever PNU 282987 was used as a stimulus, a PAM of α7-nAChR (PNU 120596) was added to the stimulation medium to increase the agonist-induced response. To check the responsiveness of the cell in regard to the [Ca^2+^]_i_ signal, the experimental protocol included a final high K^+^ depolarizing pulse. Fluorescence measurements were carried out using the microplate reader FLUOstar Optima (BMG Labtech). Wavelengths of excitation/emission were 485/520 nm and changes in fluorescence were measured at 0.5 s intervals. Once the experiment was finished, 50 μl Triton X-100 (10%) followed by 50 μl MnCl_2_ (1 mM) were added to each well to calculate maximum fluorescence (*F*_*max*_) and minimum fluorescence (*F*_*min*_), respectively. The [Ca^2+^]_i_ rise (ΔCa^2+^) induced by increasing concentrations of PNU 282987 or a high K^+^ solution was determined in each cell variant type and represents the percentage of the peak fluorescence value (% *F*_*p*_) elicited by the stimulus according to the following formula: % *F*_*p*_ = [(*F*_*p*_ − *F*_*0*_)/*F*_*max*_ − *F*_*min*_] × 100; where *F*_*0*_ is the basal fluorescence value obtained before cell stimulation. All experiments, performed in triplicate, were replicated in several independent cultures. The concentration of PNU 282987 (in μM) eliciting half-maximal ΔCa^2+^ response (EC_50_) and the slope in the concentration–response curves corresponding to each cell variant were determined by nonlinear regression using the GraphPad Prism 5 software. Student’s *t*-test was applied for statistical analysis.

### Measurement of [Ca^2+^]_i_ in single SH-SY5Y cells by fluorescence microscopy

Cells seeding on coverslips (15,000 cells/coverslip) were loaded with 4 μM of Fura 2-AM and 0.02% of pluronic acid for 45 min at 37 °C in the dark. The cells were thoroughly washed, at room temperature, with Krebs-HEPES solution for 5 min before they were placed in a perfusion chamber on the stage of an Axiovert 200 inverted epifluorescence microscope equipped with filters to excite the probe and capture its emission (XF04-2, Omega Optical). The chamber was continuously superfused, at room temperature, with Krebs-HEPES solution, and cells were stimulated with a fixed concentration of the selective α7-nAChR agonist, PNU 282987 (+PAM). The experimental design included a final high K^+^ depolarizing pulse as a guarantee of cellular functionality. Solutions containing PNU 282987 or high K^+^ were changed using a multibarreled concentration-clamp device that electrically controled the valves. Only one experimental protocol was run on each coverslip. Single-cell fluorescence measurements were performed by exciting the Fura-2-loaded cells with alternating 360- and 380 nm filtered light. The emitted fluorescence was filtered through a 520 nm filter and captured with an ORCA-R2 CCD camera (Hamamatsu Photonics). The data were acquired and stored using HCImage software and exported to Igor Pro (WaveMetrics) to perform analysis. The fluorescence values induced by the different stimuli were calculated after subtracting the basal fluorescence obtained before cell stimulation. The [Ca^2+^]_i_ signal was expressed by the ratio of fluorescence at 360 nm and 380 nm. The increase in the fluorescent signal generated by the α7-nAChR’ agonist or by the high K^+^ solution represented the change in [Ca^2+^]_i_ induced in real time by both stimuli. The kinetic parameters analyzed were: Peak Amplitude (F360/F380 ratio at the peak of fluorescence), Area Under Curve (or integral over the deviation throughout the transient [Ca^2+^]_i_ signal), Time to Peak (time between the application of the stimulus and the fluorescence peak), and Decay Time (time constant for the exponential fit to the decay curve during recovery of the basal [Ca^2+^]_i_ signal). The Kruskal–Wallis test followed by the Dunn post-hoc test were used for statistical analysis.

### Measurement of exocytosis in single SH-SY5Y cells using the FM1-43 dye

Cells seeded on coverslips (15,000 cells/coverslip) were mounted in the perfusion chamber on the stage of an Axiovert 200 inverted microscope equipped with a 63x PlanNeofluar immersion oil objective. Synaptic vesicles were loaded with the fluorescent FM1-43 membrane dye (4 μM, 4 min) by substitution of the basal Krebs-HEPES solution in the perfusion with a Krebs-HEPES solution containing 100 mM [K^+^] (prepared by equimolar replacement of NaCl by KCl). Next, cells were washed for 10 min with the basal solution to remove the excess FM1-43. Cells were subsequently subjected to two successive stimuli of PNU 282987 (+PAM) and high K^+^ (100 mM) to induce the exocytosis of the vesicles labeled with the probe, which was deduced by the loss of the recorded fluorescent signal. Exocytosis of the probe in response to the stimulus was determined by successive captures of images taken in regions of interest (ROI), at 0.5 Hz intervals and 200 ms per capture, using a high-resolution ORCA-R2 CCD camera (Hamamatsu Photonics). Data were acquired and stored using the HCImage software (Hamamatsu Photonics). Net fluorescence in each ROI at a given time was calculated by subtracting the background fluorescence in an adjacent ROI of the same cell at that time. The destaining traces were graphed as the change in fluorescence (ΔF, absolute values) normalized to the fluorescence at the beginning of the experiment (F_0_) (considered as 1 or 100, depending on the representation) using the following equation: ΔF/F_0_ = (F_F_ − F_0_)/F_0_, where F_F_ is the point at which the destaining curve slope becomes zero (complete discharge of the previously endocytosed probe). Differences between groups were evaluated for statistical significance using the Kruskal–Wallis test followed by the Dunn post-hoc test. Statistical analysis was performed using the GraphPad Prism software.

### Silencing endogenous dupα7 gene expression with siRNAs

SH-SY5Y cells were seeded in 6-well plates (625,000 cells/well) and transfected with three different siRNAs designed to knockout *dupα7* gene expression or with a siRNA control using the *Trans*IT-TKO siRNA transfection reagent (Mirus Bio) according to the manufacturer’s protocol. Since the α7 and dupα7 transcripts are highly homologous (>99%) from exon 5 to the 3'-UTR region, the target sequences for the three siRNAs used in this study were located in the divergent region (exons D, C, B and A) of the dupα7 mRNA sequence that has replaced exons 1 to 4 of the parent *CHRNA7* gene (encoding α7 mRNA). Thus, the sequences of siRNA primers and location of the target sequences (in parentheses) in the dupα7 mRNA sequence (NM-139320.1) were the following: siRNA-1, sense: 5’-CAACAUUAAGAUUACAAGUTT-3’ and reverse: 5’-ACUUGUAAUCUUAAUGUUGCG-3’ (junction region between exons D and C); siRNA-2, sense: 5’-CGGUGGAGUCGGUUAUAAATT-3’ and reverse: 5’-UUUAUAACCGACUCCACCGAC-3’ (exon D); and siRNA-3, sense: 5’-CCAUUAUUGACAAUCCAAATT-3’ and reverse: 5’-UUUGGAUUGUCAAUAAUGGGG-3’ (exon C). Data were expressed as means ± SD using the Kruskal–Wallis test followed by the Dunn post-hoc test for statistical analysis.

### Determination of dopamine release by ELISA

DA released exocytotically into the extracellular medium after stimulation of SH-SY5Y cells was determined using an ELISA kit that detects this neurotransmitter with high sensitivity (lower detection limit of 4.53 pg/ml). To amplify the DA released by the stimulus, cells seeded in 6-well plates (500,000 cells/well) were incubated with exogenous DA (20 nM) for 20 min at 37 °C in order to increase the vesicular content of neurotransmitter. After exposure to the stimulus, the cells in each well were lysed to determine the protein concentration using a BCA protein assay kit, while the corresponding supernatant was collected in a tube containing a stabilizing solution that was immediately frozen at −80 °C until the DA concentration was quantified in a microtiter plate reader (FluOstar Optima, BMG) at 450 nm using a standard curve of known concentrations of exogenous DA. The concentration of DA released in response to a specific stimulus in each cell variant assayed was normalized to the cell protein content and expressed in pg/mg of protein. Differences between groups were evaluated for statistical significance using the one-way ANOVA followed by Tukey post-hoc comparison tests.

## Data availability

All relevant data are contained within the article.

## Conflict of interest

The authors declare that they have no conflicts of interest with the contents of this article.
